# Evaluation of different radon guideline values based on characterization of ecological risk and visualization of lung cancer mortality trends in British Columbia, Canada

**DOI:** 10.1186/s12889-015-2438-2

**Published:** 2015-11-19

**Authors:** Michael C. Branion-Calles, Trisalyn A. Nelson, Sarah B. Henderson

**Affiliations:** Spatial Pattern Analysis and Research Laboratory, Department of Geography, University of Victoria, Victoria, Canada; Environmental Health Services, BC Centre for Disease Control, Vancouver, Canada; School of Population and Public Health, University of British Columbia, Vancouver, Canada

**Keywords:** Radon, Radon mapping, Radon concentration thresholds, Lung cancer, Prevention

## Abstract

**Background:**

There is no safe concentration of radon gas, but guideline values provide threshold concentrations that are used to map areas at higher risk. These values vary between different regions, countries, and organizations, which can lead to differential classification of risk. For example the World Health Organization suggests a 100 Bq m^−3^value, while Health Canada recommends 200 Bq m^−3^. Our objective was to describe how different thresholds characterized ecological radon risk and their visual association with lung cancer mortality trends in British Columbia, Canada.

**Methods:**

Eight threshold values between 50 and 600 Bq m^−3^ were identified, and classes of radon vulnerability were defined based on whether the observed 95^th^ percentile radon concentration was above or below each value. A balanced random forest algorithm was used to model vulnerability, and the results were mapped. We compared high vulnerability areas, their estimated populations, and differences in lung cancer mortality trends stratified by smoking prevalence and sex.

**Results:**

Classification accuracy improved as the threshold concentrations decreased and the area classified as high vulnerability increased. Majority of the population lived within areas of lower vulnerability regardless of the threshold value. Thresholds as low as 50 Bq m^−3^ were associated with higher lung cancer mortality, even in areas with low smoking prevalence. Temporal trends in lung cancer mortality were increasing for women, while decreasing for men.

**Conclusions:**

Radon contributes to lung cancer in British Columbia. The results of the study contribute evidence supporting the use of a reference level lower than the current guideline of 200 Bq m^−3^ for the province.

## Background

Radon is a colourless, odourless, radioactive noble gas produced by the breakdown of naturally occurring uranium within the surface of the Earth. Radon is estimated to be a factor in over 3000 lung cancer deaths in Canada per year [1]. Radon atoms can be transported from their source and into homes where concentrations can accumulate. The dose–response relationship between radon exposure and lung cancer risk is understood to be linear, with no evidence of a threshold [2–5]. As such, there is no radon concentration at which there is no risk of developing lung cancer, and the probability of developing lung cancer increases with exposures to higher concentrations. Individuals who smoke are at an even greater risk due to the synergistic effects of radon and cigarette smoke [6]. Although the linear no-threshold model has been disputed [7], it is accepted within the Canadian radiation protection policy set by the Canadian Nuclear Safety Commission. These regulations implement the principle of ALARA, which states that public radiation exposures and doses should be kept “as low as reasonably achievable” [8]. This study follows the principle of ALARA, as is consistent with Canadian policy.

In light of the public health threat posed by residential radon, varying concentration thresholds have been set by different regions, countries, and organizations throughout the world. Here we define a threshold value as the concentration above which remedial action to reduce radon is recommended. These thresholds do not imply a level of safety, but rather a concentration below which the risk of developing radon-induced lung cancer is considered acceptably small. Threshold values are chosen to maximize the overall reduction in lung cancer mortality while considering what is practical to achieve in a majority of homes in a given jurisdiction [1]. Though the World Health Organization (WHO) recommends a concentration threshold of 100 Bq m^−3^, other established thresholds are typically higher. For example, the USA uses a threshold of 148 Bq m^−3^, Canada uses a threshold of 200 Bq m^−3^, and the European Union uses thresholds ranging between 200 and 400 Bq m^−3^ [9, 10].

Radon concentration thresholds are used to inform policy and to enable risk communication. For example, radon risk maps characterize the ecologic radon risk associated with indoor radon by identifying spatial areas more prone to high radon concentrations. Such maps allow for geographic targeting of radon awareness, testing, and remediation campaigns, and they can also encourage new policies [11]. The radon risk map of Ireland divided the country into grid squares and mapped the proportion of homes whose indoor concentration exceeded the national threshold of 200 Bq m^−3^ [12]. Those grid squares where >10 % of homes were estimated to exceed the national threshold were designated as high radon areas (HRAs). After completion of the map, an updated building code required that all new buildings be fitted with a standby radon sump that could be installed at a later date. Buildings within the HRAs were required to install a radon barrier in addition to the standby sump [12]. The choice of threshold concentration for use in such mapping is generally based on the recommended threshold used in the geographic jurisdiction for which the map is being prepared. However, the choice of threshold will affect the size of the spatial area classified as high risk and any resulting policy, and it may affect the accuracy of the classification. If the concentration threshold in Ireland was higher or lower than 200 Bq m^−3^ it would have changed the designation of HRAs and the requirement of additional radon protection measures in new buildings.

Ultimately, the objective of any radon risk map is to effectively delineate areas at risk of high indoor radon concentrations and, therefore, greater rates of radon-induced lung cancer. Temporal trends in the annual crude ratio of lung cancer mortality can be used as an exploratory tool for investigating spatial differences in radon distribution [13]. As such, we expect that an effective radon risk map would show distinct differences in lung cancer mortality trends between regions defined as higher and lower risk. However, the delineation of higher and lower risk areas depends on the chosen concentration threshold.

Our objective is to evaluate how different radon concentration thresholds are associated with the accuracy of ecological radon risk classification, geographic areas classified as higher or lower radon risk, populations classified as higher or lower risk, and visual temporal trends in lung cancer mortality. Understanding these relationships has important implications for informing policy on appropriate guideline values. Following Branion-Calles et al. (2015) we map the radon vulnerability of geologic units using eight thresholds ranging from 50 to 600 Bq m^−3^. Radon vulnerability refers to the potential for a geographic area to exceed a specified concentration threshold. Maps of indoor radon vulnerability are then used to visually explore the association between radon concentration thresholds and lung cancer mortality trends stratified by sex and smoking prevalence. This is not intended nor designed to be an epidemiologic study of the association between radon exposure and lung cancer, but we use ecologic information about both to assess the implications of different radon thresholds from an environmental health policy perspective. All data employed in this study are archived at the BC Center for Disease Control (BCCDC) and are not publicly available, except where indicated.

## Methods

### Study area

**Fig. 1:**
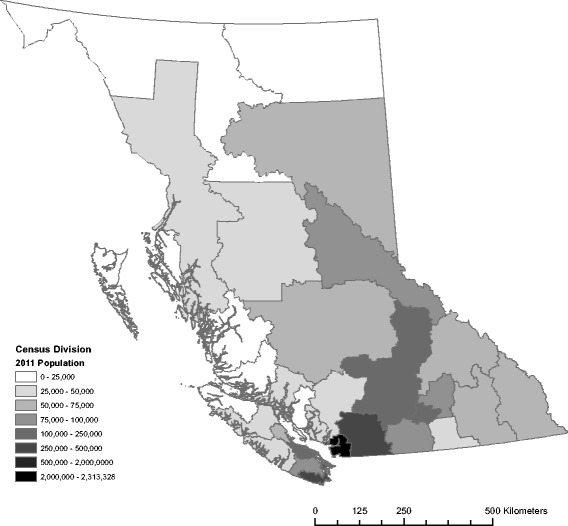
The study area of British Columbia, Canada. The spatial distribution of the provincial population by census division boundaries is shown

The study area was the province of British Columbia (BC), on the west coast of Canada. Many parts of BC are prone to high radon concentrations, including both small and large communities, primarily within the interior and northern regions [13–15]. In the 2011 census BC had a population of approximately 4.4 million people*,* with 3.79 million living in urban areas and 609,000 living in rural areas. The majority of the population lives within a small area in the southwestern region (Fig. 1).

### Bedrock dissemination areas

The province was divided into 36,061 mapping units based on an intersection of census dissemination areas and simplified bedrock lithology. Each mapping unit was labelled as a “Bedrock Dissemination Area” (BDA) and was assumed to represent a homogenous spatial area with respect to the environmental and housing conditions that would affect its potential for high indoor radon concentrations. In order to enable the classification of indoor radon risk each BDA were associated with variables derived from overlapping geospatial datasets including: indoor radon concentration data, geologic, soil, meteorological, hydrological and neighbourhood housing data.

### Bedrock dissemination areas - indoor radon concentrations and vulnerability class

Indoor radon concentration data are archived at the BCCDC and consist of five disparate surveys conducted between 1991 and 2014. Surveys were conducted by the BCCDC, the Northern Health Authority, the BC Lung Association, the Donna Schmidt Foundation and a private contractor. The BCCDC survey consisted of two surveys, the first of which was designed to oversample in areas with known high ambient radiation levels and the second oversampled in areas with moderate ambient radiation levels. The remaining four surveys collected measurements through volunteers. Attributes common to each survey were a six digit postal code, date of test period and the observed radon concentration. Each indoor radon concentration observation was assigned a geographic coordinate based on its associated six digit postal code and date through geocoding. A total of 4352 indoor radon concentrations were successfully assigned a spatial location.

**Fig. 2:**
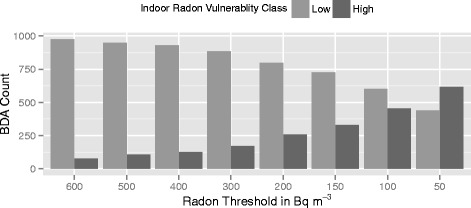
The class distribution of bedrock dissemination areas (BDAs) in the training dataset using each threshold value

Indoor radon concentration values were used to construct the response variable for the purposes of statistical classification. We used the same classification of indoor radon risk, termed indoor radon *vulnerability*, developed in previous work [14]. Indoor radon vulnerability refers to a way of characterizing ecologic risk, where classes of risk are assigned based on whether the observed 95^th^ percentile concentration was above or below a specified threshold. In this way indoor radon vulnerability classification describes the relative potential for homes to have a concentration higher than the threshold value. Multiple binary response variables were defined where each BDA with observed concentrations was assigned an indoor radon vulnerability classification based on the following thresholds: 50, 100, 150, 200, 300, 400, 500, and, 600 Bq m^−3^. These values were selected based on the premise that they cover the range of radon threshold concentrations used in countries throughout the world and represent multiple scenarios of provincial radon risk. Of the total 36,051 BDAs, there were 1054 that contained at least one indoor radon measurement. These BDAs comprised the training dataset, leaving the remaining 34,972 BDAs to be classified using model results*.* A binary indicator of either high or low vulnerability was assigned to each BDA in the training dataset based on whether the observed 95^th^ percentile radon measurement was greater or less than each concentration threshold. This resulted in eight different class distributions for the training dataset (Fig. 2).

### Bedrock dissemination areas - independent variables

The potentially predictive independent variables were selected based on their theoretical association with local radon concentrations, either individually or in combination. For example, soils that allow for a greater rate of radon transport towards the subsurface may increase the quantity of radon available to be transported into homes [16–18]. Similarly, colder ambient temperatures may increase the difference between indoor air and outdoor air and therefore increase the rate at which soil gas is drawn indoors [19]. The transport of radon into homes can be further affected by specific housing characteristics, such as cracks in the foundation and the ventilation rate [20]. Although we did not have such data about the individual homes, we do have neighbourhood data on average home age and state of repair from the 2011 National Household Survey [21].

The specific independent variables we constructed for each BDA were each derived from publicly available data and include: (1) simplified bedrock lithological class from the BC Digital Geology Open File (BCDGOF) [22]; (2) geologic fault presence from the BCDGOF [22] ; (3) dominant soil parent material from the Soil Landscapes of Canada Version 2.2 (SLC) [23]; (4) dominant soil drainage class from the SLC [23]; (5) dominant rooting depth class from the SLC [23]; (6) dominant soil coarse fragment content from the SLC [23]; (7) dominant kind of surface material from the SLC [23]; (8) average winter temperature (climate normals) from the Climate Western North America database (CWNA)[24]; (9) average winter precipitation (climate normals) from the CWNA[24]; (10) distance to nearest major river from the Freshwater Atlas of BC [25]; (11) dominant age of home from the 2011 National Household Survey of Canada (NHSC) [21]; (12) proportion of homes in need of major repairs from the 2011 NHSC [21]; and (13) distance to nearest uranium mineralization. The last variable was not included our previous work [14], but homes built on materials with high uranium content may be more prone to higher radon concentrations [20]. Distance to nearest uranium mineralization was obtained by calculating the Euclidean distance from spatially referenced locations of known mineral occurrences with a significant quantity of uranium. Mineral occurrence data in British Columbia are available from the British Columbia Ministry of Energy and Mines [26]. Each mineral occurrence in the database had a spatial location as well as a description of the present elements or substances that had economic potential. Detailed rationale and methods for the other 12 variables is given elsewhere [14].

### Bedrock dissemination areas - population estimates

Estimates of the resident population for each BDA were made using data from the Dissemination Area (DA) level of the 2011 national census which are publicly available [27]. These spatial areas generally include between 400 and 700 persons. Because BDAs represent the intersections between DAs and the bedrock geography, BDAs are smaller than their parent DAs. The population of a BDA was therefore estimated based on the proportion of its total area relative to the area of its parent DA. For example, if a 2 km^2^ DA had 500 residents and it was split into two 1 km^2^ BDAs, each would be assigned an estimated population of 250.

### Mortality records

Mortality records provided by the provincial Vital Statistics agency are archived at the BCCDC. These data include information about age, sex, underlying cause of death, and postal code of residence for each decedent. The underlying cause of death is coded according to the International Classification of Diseases 10^th^ Revisions (ICD-10). We extracted deaths due to all natural causes (excluding ICD-10 codes starting with T through Y) and lung cancer (ICD-10 code C34) for adults aged 20 and over from 1998 through 2013. Each death was anonymously mapped by geocoding its residential 6-digit postal code.

### Smoking prevalence

There are 89 local health areas (LHAs) in BC, and these are the smallest spatial area at which health services are administered. Data on smoking prevalence were available at the LHA level from the BC Ministry of Health, which contracted Statistics Canada to oversample in BC during the 2008–2009 Canadian Community Health Survey [28, 29]. Data from some of the smaller LHAs were combined to ensure statistical validity, resulting in 83 rather than 89 estimates. Each LHA was assigned a binary classification of higher or lower smoking based on whether its smoking prevalence was above or below the median of all 83 estimates.

### Indoor radon vulnerability modelling and mapping

Following the methods outlined in Branion-Calles et al. (2015) we used a balanced random forest algorithm to classify radon vulnerability based on whether model estimates were above or below the eight threshold values (50, 100, 150, 200, 300, 400, 500, and 600 Bq m^−3^). Indoor radon concentrations result from a complex combination of environmental and housing characteristics and therefore necessitate a modelling technique that can capture this complexity [17]. Random forests are used to model complex environmental processes because they are a non-parametric ensemble classifier with a high predictive ability and the flexibility to accommodate mixed variable types, non-linear relationships, and high order interaction effects [30, 31]. The random forest algorithm works by combining the results of a user specified amount of maximally grown classification trees. Each classification tree is created by randomly selecting a bootstrapped sample of the training data and continually splitting the sample into two subsets based on the value of an independent variable, until all subsets can no longer be split. For each split the algorithm first selects a random subset of all available independent variables and, second, searches all possible binary splits based on the whole range of values within the selected subset of independent variables. The split that is chosen maximizes the class homogeneity within each resulting subset. When results are aggregated over all trees, the variability between trees in the forest reduces over-fitting and susceptibility to outliers in the model [30, 31]. The balanced approach modifies the random forest algorithm by ensuring that there is equal representation in each bootstrapped sample from which each tree is grown in order to more effectively classify the minority class in an imbalanced dataset [32].

Estimates of predictive accuracy can be made without an independent validation dataset by using “out-of-bag” (OOB) data. This refers to the observations that were left out of any given bootstrapped sample [33]. In order to obtain an unbiased estimate of predictive performance, the OOB observations for each classification tree are dropped down and assigned a predicted classification. The final prediction for each observation is given based on its majority classification over all trees for which it was OOB. For all observations the OOB prediction can be compared with its observed class to derive an unbiased estimate for the predictive performance of the model through an assessment of the so-called confusion matrix.

Class accuracy, class precision, and a kappa statistic can all be generated from the OOB confusion matrix to evaluate model performance. Class accuracy refers to the proportion of a given observed class that was correctly classified. Class precision refers to the proportion of the given predicted class that were correctly classified. A kappa statistic quantifies the improvement of the classifier compared with a random classifier, which can be a robust measure to evaluate overall classifier performance for imbalanced datasets [34].

An individual balanced random forests model was trained on the subset of 1054 BDAs that had observed vulnerability classes based on the eight selected radon thresholds [32, 33]. Tests for spatial autocorrelation in both the original radon data (Geary’s *c* = 0.86, *p* = 0.2) and the 95^th^ percentile aggregated (BDA units) data (Geary’s *c* = 0.89, *p* = 0.186), indicate no significant spatial autocorrelation and suggest spatial independence of observations. To ensure stable results each model combined twenty balanced random forest algorithm runs consisting of 10,000 individual classification trees. The model performance was compared by evaluating class accuracy, class precision, and kappa scores. A vulnerability classification was assigned to the unmeasured BDAs from each model, resulting in eight different maps. Approximately 23 % of BDAs in the province had independent variable values not contained within the training dataset, which made them ineligible for prediction. For each map the regional differences in vulnerability were assessed by comparing (1) the geographic areas classified as high and (2) the number of people living within those areas, where regions were defined by census division boundaries [35].

### Visual comparison with lung cancer mortality trends

The visual relationship between radon thresholds and temporal lung cancer mortality trends was assessed by comparing the annual ratio of lung cancer mortality to all natural mortality in high and low vulnerability regions. Each death was spatially assigned to a radon vulnerability class for each of the eight predictive maps. Additionally, each death was assigned to higher or lower smoking prevalence based on the LHA in which it occurred. By attributing each death with both radon and smoking classifications we were able to compare trends across high and low radon vulnerability while crudely accounting for the confounding factor of smoking prevalence. For each radon concentration threshold, the annual sum of lung cancer deaths was divided by annual sum of all natural deaths in the high and low vulnerability areas. The values for 1998 through 2013 were plotted, and a temporal trend line was fitted using a locally weighted LOESS smoother. The same was done to explore potential differences between males and females, as was previously observed in BC [13].

## Results

### Indoor radon vulnerability modelling and mapping

The difference in overall classification accuracy between models improved as the radon threshold decreased (Table 1). The Kappa score for each model improved with each reduction in concentration threshold. The greatest gains in performance as measured by Kappa were found in the reductions from 600 to 500 Bq m^−3^, 300 to 200 Bq m^−3^, and 150 to 100 Bq m^−3^, with gains of 0.11, 0.08, and 0.11, respectively. Reductions from 500 to 300 Bq m^−3^, 200 to 150 Bq m^−3^, and 100 to 50 Bq m^−3^ resulted in minimal improvement to the Kappa score.

**Table 1:** The classification metrics for each balanced random forest algorithm. Accuracy is defined as the proportion of an observed class that was correctly classified. Precision is defined as the proportion of a predicted class that was correctly classified. Kappa can be interpreted as the percent improvement in overall accuracy of a classifier compared with the expected overall accuracy of a random classifier

	Threshold in Bq m^−3^	Lower-than-threshold Accuracy	Lower-than-threshold Precision	Higher-than-threshold Accuracy	Higher-than-threshold Precision	Kappa	Kappa Gain
**a)**	600	0.81	**0.97**	0.69	0.22	0.25	0
**b)**	500	**0.83**	0.96	0.72	0.32	0.36	**0.11**
**c)**	400	**0.83**	0.96	0.74	0.37	0.39	0.03
**d)**	300	0.8	0.94	0.73	0.42	0.41	0.02
**e)**	200	0.8	0.91	0.76	0.55	0.49	0.08
**f)**	150	0.77	0.88	0.76	0.6	0.5	0.01
**g)**	100	0.79	0.86	0.83	0.75	0.61	**0.11**
**h)**	50	0.77	0.8	**0.86**	**0.84**	**0.63**	0.02

Values in bold indicate the highest value between threshold models

Models for lower radon thresholds were better able to accurately the predict the high vulnerability classification, which lead to the observed gains in the Kappa score. Estimates of the accuracy for high vulnerability classification increased from 0.69 to 0.86. Class precision also increased with each successive reduction in radon threshold, from 0.22 to 0.84. Conversely, estimates for the accuracy of low vulnerability classification decreased from 0.83 to 0.77 and estimates of its class precision decreased from 0.97 to 0.8 (Table 1). The gains in class accuracy and class precision for the high vulnerability class with the use of a lower concentration threshold were much greater than the decreases in class accuracy and class precision in the low vulnerability class.

**Fig. 3:**
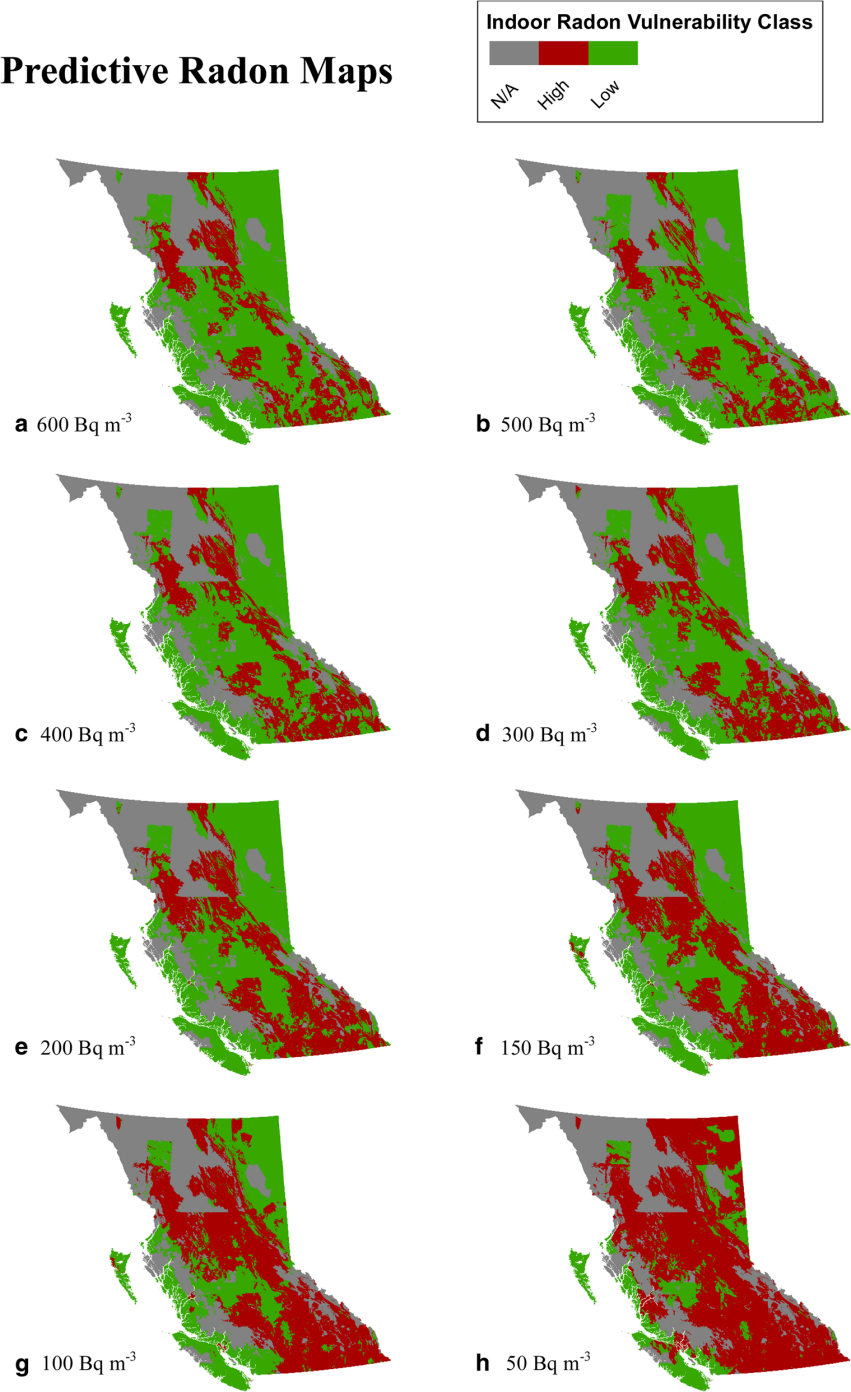
Estimated vulnerability maps for each of the eight radon threshold. Red areas indicate high vulnerability, green areas indicate low vulnerability, and grey areas indicate regions without adequate data for modelling

**Fig. 4:**
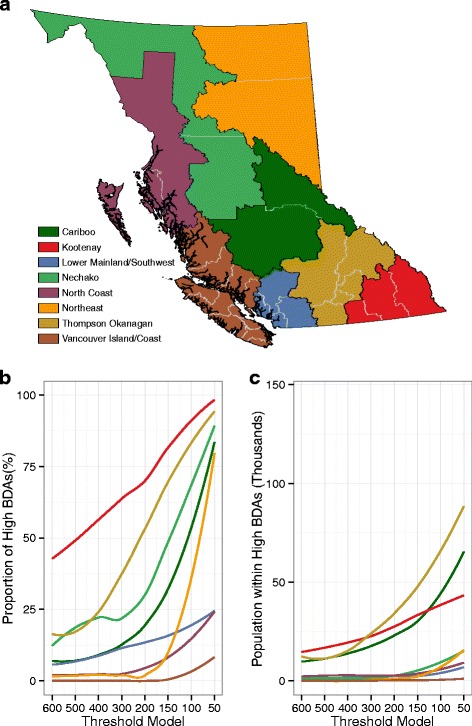
Changes in regional vulnerability classification based on changes in threshold values plotted by the proportion of high BDAs by census division (**b**) and the estimated population living within high BDAs (**c**). The colours all correspond to the legend in (**a**). Census divisions are demarcated by grey lines in (**a**), and they aggregate up to the coloured economic regions (**a**). Trends in (**b**) and (**c**) were fitted using a locally-weighted LOESS smoother

The overall provincial prevalence of high vulnerability areas increased with lower concentration thresholds (Fig. 3), but some regions were more affected than others. Census divisions in the central and northeast had the largest increases across decreasing radon thresholds, but the highly populated southern coastal areas were generally not affected. The relative ranks of areas at risk were minimally affected by changes in concentration thresholds. For example, census divisions within the Kootenay economic region were at highest risk across all thresholds. The Northeast economic region was most affected, showing a rapid increase in high vulnerability with decreasing threshold values (Fig. 4).

The total number of residents living in high vulnerability areas increased as the concentration threshold decreased, but the rate of increase varied regionally. Census divisions within the Thompson Okanagan, Cariboo, and Kootenay economic regions consistently had higher numbers of residents living in high vulnerability areas compared with the rest of the province, regardless of the threshold. Although there were large increases in the geographic area classified as high vulnerability in the Northeast and Nechako economic regions, the number of people living in those areas remained low due to their sparse populations (Fig. 4).

### Lung cancer mortality trends

**Fig. 5:**
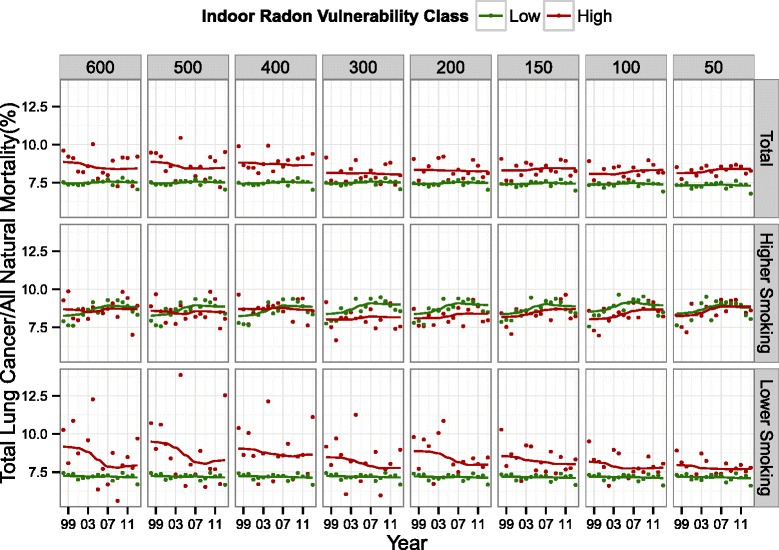
The annual ratio of lung cancer mortality to all natural mortality (the crude lung cancer mortality ratio) within high and low vulnerability areas plotted from 1998–2013 for each predictive map based on eight threshold values. The columns show the threshold values in Bq m^−3^, which were used to delineate low and high vulnerability. The rows show the total trends, and the trends when stratified by higher smoking LHAs and lower smoking LHAs. The lung cancer mortality trends were fitted with a locally-weighted LOESS smoother

The total number of natural and lung cancer deaths occurring in high radon vulnerability areas increased with decreasing radon thresholds, but the ratio of the proportions between the groups remained stable (Table 2). The trends for the entire province showed that areas with high radon vulnerability consistently had higher proportions of lung cancer mortality across all radon thresholds (Fig. 5). Further, there was little change in the distance between the high and low vulnerability lines with decreasing radon thresholds. When plots were stratified by higher and lower smoking prevalence there was no clear separation between the lung cancer trends in areas with higher smoking. However, in areas with lower smoking prevalence, the high radon vulnerability areas had consistently higher proportions of lung cancer mortality, and the separation between lines decreased as the radon threshold decreased. The trends in lung cancer mortality for low radon vulnerability areas with lower smoking were flat and stable at ~7.5 % for all thresholds while the trends in higher smoking areas were curved and increasing (Fig. 5).

**Table 2:** The number of deaths during the study period (1998–2013) due to lung cancer and all natural causes stratified by the ecologic exposure variables assigned to each mortality by geographic location including smoking prevalence (high) and radon vulnerability (high)

	Threshold in Bq m^−3^	All Natural Deaths (% of Total)	Lung Cancer Deaths (% of Total)	Ratio of the percentages
Total (N)		457,242	34,443	
Higher Smoking		22.9 %	26.2 %	1.14
High radon	600	3.3 %	3.7 %	1.12
High radon	500	3.2 %	3.7 %	1.16
High radon	400	4.2 %	4.8 %	1.14
High radon	300	6.5 %	7.0 %	1.08
High radon	200	8.6 %	9.5 %	1.10
High radon	150	11.1 %	12.4 %	1.12
High radon	100	16.5 %	18.0 %	1.09
High radon	50	22.2 %	24.4 %	1.10

**Fig. 6:**
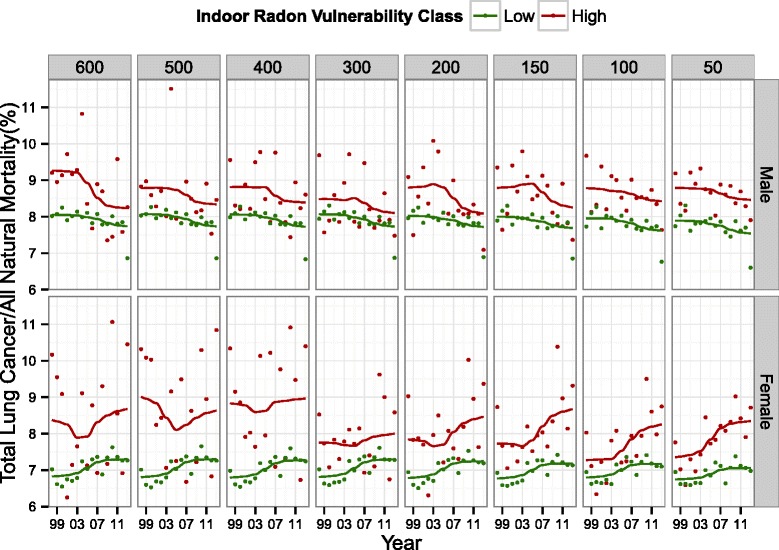
The annual ratio of lung cancer mortality to all natural mortality (the crude lung cancer mortality ratio) within high and low vulnerability areas plotted from 1998–2013 for each predictive map based on eight threshold values. The columns show the threshold values in Bq m^−3^, which were used to delineate low and high vulnerability. The rows show the trends stratified by sex

When lung cancer mortality trends in high and low vulnerability areas were stratified by sex, high vulnerability areas were consistently associated with higher proportions of lung cancer mortality across radon thresholds for both males and females. For each threshold, the trend lines for males in both high and low vulnerability areas showed a slight decrease through time while they were increasing through time for females. The ratios for females in high vulnerability appeared unstable for thresholds greater than 300 Bq m^−3^ (Fig. 6).

## Discussion

Different regions, countries, and organizations recommend different radon concentration thresholds that essentially classify the associated risk of lung cancer as being acceptable or unacceptable. In reality, however, radon is a non-threshold carcinogen and any level of exposure carries some risk [2]. Established guideline concentration values reflect a balance between the health evidence, what is practically achievable, and other political and public health priorities. Many epidemiologic studies have examined the association between radon exposure and lung cancer, but there has been little systematic evaluation of how decisions about guideline values affect important policy considerations. These include the accuracy with which risk can be classified, the extent of geographic areas classified as high risk, the size of the populations classified as high risk, and the observed relationships between risk areas and lung cancer mortality trends. Here we have addressed this gap by exploring the impacts of thresholds ranging from 50 to 600 Bq m^−3^ in one Canadian province with previously demonstrated spatial variability in radon risk [13, 14, 36].

We found that the accuracy of risk classification was improved as the threshold decreased, likely due to increasing balance of the training data. Though the balanced random forest algorithm is more effective at classifying imbalanced datasets than an unmodified random forest, it is still designed to minimize the overall error. This appeared to be more effective when high and low vulnerability were delineated using a lower threshold, resulting in a more balanced dataset [32]. Due to the potential for misclassification of individual BDAs, each threshold map should be interpreted at regional scale rather than at the individual mapping unit.

Unsurprisingly, the geographic extent of areas classified as high risk became larger as the thresholds decreased. However, much of BC is sparsely populated, so it was more important to consider changes in the populations classified as high and lower vulnerability as the thresholds changed. The number of people living in high vulnerability areas increased from approximately 326,000 to 824,800 when the threshold was reduced from the current guideline value of 200 Bq m^−3^ to the minimum value of 50 Bq m^−3^. Given that high vulnerability areas were associated with higher prevalence of lung cancer mortality, the increase in exposed population indicates that adoption of a higher threshold value has the potential to mask some risk.

Regardless of threshold employed, the majority of the provincial population lived in areas classified as low vulnerability. At the current guideline value of 200 Bq m^−3^ only 7 % of BC residents were estimated to live in areas of high vulnerability. However, there is direct evidence that indoor radon concentrations contribute to lung cancer mortality in the general population at concentrations less than the Health Canada guideline [2]. A study in the UK estimated that approximately 96 % of radon-related lung cancer deaths resulted from exposures to indoor concentrations less than 200 Bq m^−3^, due to the large number of people exposed to these lower risk concentrations [37]. Given that the majority of the BC population is exposed to concentrations lower than 200 Bq m^−3^ it is likely the majority radon-related lung cancer deaths result from exposures less than 200 Bq m^−3^.

The crude lung cancer mortality ratio within areas classified as high vulnerability was higher than within areas classified as low vulnerability for every concentration threshold through time. However, the distance between high and low vulnerability lines was consistent across thresholds, possibility indicating the presence of a confounding variable. Smoking rates are the primary predictor of population-level lung cancer risk, which results in geographic variations in lung cancer mortality trends being dominantly associated with geographic variations in smoking prevalence [38–40]. In areas with higher smoking prevalence, we observed no differences in lung cancer mortality trends between high and low vulnerability areas. In areas with lower smoking prevalence, however, differences between radon vulnerability areas were clear across all threshold values. The distance between trend lines decreased with lower thresholds, possibly indicating some ecological control of confounding effects of smoking rates. Radon and smoking have a synergistic relationship at the individual level [6, 41], but radon vulnerability appeared to have little effect on population mortality trends in high smoking areas. Furthermore, the difference in trends between males and females suggests that sex may have a modifying effect on lung cancer mortality ratios in the province [13]. The difference in trends between high and low vulnerability in areas with a lower smoking prevalence suggests that the methods first developed in Branion-Calles et al. (2015) were able to delineate areas of higher and lower radon risk.

Although this approach was designed to evaluate policy options with respect to radon guideline values, it has important limitations from an epidemiologic perspective. First, both radon and smoking categories were assigned ecologically, based on the geographic area of residence, meaning that most individuals were misclassified. For example, 100 % of decedents who lived in an area with 5 % of homes over the threshold value were classified as having high radon exposure. Although this approach is crude, experts argue that it can be useful in studies where geographic differences drive population variability in exposure [42]. Second, we did not consider the life course exposure to radon and other environmental or occupational lung carcinogens [43] in these analysis, which were conducted with secondary administrative data. The residential and occupational histories of all decedents were unknown, thereby excluding the possibility of accounting for migration of populations during the latency period of lung cancer formation.

Given these limitations, it is important to consider how the visualisations of lung cancer mortality in the province stratified by radon may be biased or confounded. Bias would result in the ratio of lung cancer mortality to all natural mortality appearing to be higher or lower than in reality. For example, consider a situation in which we observe 7 % lung cancer mortality in the low radon group and 10 % in the high radon group. First, let us assume that there is no misclassification in the low radon group, but that many decedents in the high radon group actually had low radon exposure. Second, let us assume that a smaller proportion of the lung cancer cases has been misclassified compared with the deaths from all natural causes given that radon is known risk factor for lung cancer. If 90 % of all lung cancer cases were misclassified and 95 % of all other natural deaths were misclassified (because we used the 95^th^ percentile values to establish the categories), the mortality ratio in the low group would actually be 8 % compared with 22 % in the high group. On the other hand, confounding would occur if the differences that appeared to be the result of exposure to radon were actually due to another factor associated with both lung cancer and ecologic radon exposure. For example, both radon exposures and smoking prevalence are higher in the rural areas of BC than in urban centres. When we stratified analyses by geographic smoking prevalence to evaluate whether the relationship between radon and lung cancer mortality could be observed in both higher and lower smoking areas, we found that it could not be observed in areas of higher smoking prevalence. This result is consistent with previous work demonstrating the confounding between ecologic measures of radon exposure and smoking [44].

The Health Canada radon guideline is a threshold value that provides a frame of reference for making informed decisions about radon testing and remediation, but Canadian residential radon values are not regulated [45, 46]. In the absence of binding federal policy, provincial governments have the authority to independently enact radon protection legislation through changes to provincial building codes [47]. Although BC has adopted radon mitigation measures for newly constructed buildings in its provincial code, there is no legal requirement for new buildings to test below a specific concentration threshold [47]. Based on the results of our study and the principle that no radon concentration is safe, we contribute evidence surrounding the discussion of implementing a lower concentration threshold than the 200 Bq m^−3^ value currently employed by Health Canada. Though further research is needed to quantify the absolute number of lung cancer deaths related to indoor radon across the province, a lower threshold value may have the potential to reduce the burden of disease attributable to radon, especially if it was legally enforced for new buildings. While such measures would not affect the existing building stock, they would be an important step towards protecting the BC population from radon exposure in the future.

## Conclusions

We examined how different radon concentration thresholds were associated with classification accuracy, estimated areas and populations at risk, and lung cancer mortality trends in BC. Lowering the threshold from its current guideline value of 200 to 50 Bq m^−3^ resulted in better classification accuracy, a 2.5-fold increase in the relatively small population at risk, and persistent separation in lung cancer mortality trends between areas of high and low vulnerability. We suggest that it would be appropriate for BC to consider mandating a 50 Bq m^−3^ threshold value to maximize the reduction of radon-related lung cancer in the province.

## Abbreviations

BC: British Columbia
BCDGOF: BC Digital Geology Open File
BDA: Bedrock Dissemination Area
CWNA: Climate Western North America database
DA: Dissemination Area
HRA: high radon area
ICD-10: International Classification of Diseases 10^th^ Revisions
LHA: local health area
NHSC: 2011 National Household Survey of Canada
OOB: out-of-bag
SLC: Soil Landscapes of Canada Version 2.2
